# Characterizing eye gaze and mental workload for assistive device control

**DOI:** 10.1017/wtc.2024.27

**Published:** 2025-03-03

**Authors:** Larisa Y.C. Loke, Demiana R. Barsoum, Todd D. Murphey, Brenna D. Argall

**Affiliations:** 1 Department of Mechanical Engineering, Northwestern University, Evanston, IL, USA; 2 Shirley Ryan AbilityLab, Chicago, IL, USA

**Keywords:** Eye gaze, Assistive devices, Control interfaces, Workload, Characterization

## Abstract

Eye gaze tracking is increasingly popular due to improved technology and availability. In the domain of assistive device control, however, eye gaze tracking is often used in discrete ways (e.g., activating buttons on a screen), and does not harness the full potential of the gaze signal. In this article, we present a method for collecting both reactionary and controlled eye gaze signals, via screen-based tasks designed to isolate various types of eye movements. The resulting data allows us to build an individualized characterization for eye gaze interface use. Results from a study conducted with participants with motor impairments are presented, offering insights into maximizing the potential of eye gaze for assistive device control. Importantly, we demonstrate the potential for incorporating direct continuous eye gaze inputs into gaze-based interface designs; generally seen as intractable due to the ‘Midas touch’ problem of differentiating between gaze movements for perception versus for interface operation. Our key insight is to make use of an individualized measure of smooth pursuit characteristics to differentiate between gaze for control and gaze for environment scanning. We also present results relating to gaze-based metrics for mental workload and show the potential for the concurrent use of eye gaze for control input as well as assessing a user’s mental workload both offline and in real-time. These findings might inform the development of continuous control paradigms using eye gaze, as well as the use of eye tracking as the sole input modality to systems that share control between human-generated and autonomy-generated inputs.

## 1. Introduction

Humans interact with assistive technology systems through accessible control interfaces. For individuals with motor impairments, these assistive technologies can help to mitigate limitations on their independence and ability to participate in society – such as using a computer for communication or a powered wheelchair for independent mobility.

An individual’s level of motor impairment determines the types of control interfaces that they are able to effectively use. In general, the interfaces accessible to users with greater severity of motor impairment become increasingly limited in the richness of control commands that can be issued through them – including the number of simultaneous degrees of freedom that can be controlled and the resolution (discretization of the magnitude and direction) of the signal.

For example, individuals with higher levels of spinal cord injury may be unable to use a joystick, which is the most common interface for controlling powered wheelchairs. For these users, one standard alternative interface is the sip-and-puff device, which however introduces the following challenges in comparison to joystick control: It allows for the active operation of only one (of the two) wheelchair control dimensions at a time, and its signals are interpreted discretely. As a result, the physical motion of the powered wheelchair can be choppy and imprecise, and the control can be difficult to learn and mentally (and often physically) taxing (Kelliher et al., 2010).

Eye gaze is one alternative input mechanism for assistive device interfacing currently gaining traction within the field. Eye gaze trackers are especially useful for individuals with neurodegenerative diseases such as Amyotrophic Lateral Sclerosis (ALS), as voluntary eye movement is retained until the disease has progressed to terminal stages (Kanning et al., 2010). Using eye gaze trackers for communication has been shown to restore agency to ALS patients even as the motor impairment progresses – leading to improvements in quality of life (Calvo et al., 2008). A natural extension of the use of eye gaze trackers is to help restore independent mobility. However, due to reasons of safety and ease of use, current commercial eye gaze systems for controlling powered wheelchairs only give users access to discrete switch control (GazeDriver, 2022; ToltTechnologies, 2022). This limits controllability for users, which may lead to a perceived loss of user agency, in turn resulting in frustration, disuse, or even injury (Parasuraman and Riley, 1997).

High mental workload (MWL) has been identified as a factor contributing to the abandonment of assistive devices (Stassen et al., 1975; Marchand et al., 2021). Thus, it is important to understand whether ALS patients and individuals with spinal cord injuries experience high MWL while operating their control interface so as to decrease the likelihood of abandonment of the wearable assistive device. The assessment of MWL can be conducted through offline or online methodologies. Offline approaches entail subjective evaluations via post-task questionnaires, particularly valuable in comprehending end-users’ experiences with assistive technologies such as eye gaze trackers. However, due to their retrospective nature, offline measures are unsuitable for real-time MWL monitoring. Conversely, online MWL measures are characterized by objectivity and facilitate immediate adaption. However, these methods rely on physiological markers or performance indices to quantify the cognitive load experienced by users that can be invasive to record (e.g., implantable brain-computer interfaces). For these reasons, eye-tracking has emerged as a method for assessing operators’ workload, characterized by its minimal intrusiveness and robustness (Luo et al., 2021). While there is a substantial body of literature exploring the use of eye tracking to estimate mental workload (Marquart et al., 2015; Luo et al., 2023), the simultaneous use of the gaze signal for control input has not been explored.

In the field of eye-based human-computer and human-robot interaction, there has been extensive work done in the areas of eye movement event detection, human intent inference from eye motion, and usability studies of user interfaces. However, in the domain of eye-based control, the field has largely converged on eye gaze as a complementary input mode to other modalities (e.g., used together with a joystick or buttons, rather than the sole input mode (Jacob, 1990; Majaranta and Bulling, 2014; Aronson and Admoni, 2020)), and there is limited work investigating the (sole) use of eye gaze inputs for *explicit* and *continuous* assistive device or robot control. In order to design an eye-based user interface for continuous robot control, a better understanding of the characteristics of eye gaze signals when operating an eye-based input system, as well as limitations in the eye gaze signal, is necessary.

This work presents the following contributions:

1. A suite of open-source assessment and data-gathering tasks^1^ for use with an eye gaze tracking interface.
2. An open-source software system^1^ for interfacing an eye gaze tracker for real-time control, integrated within the Robot Operating System (ROS) (Macenski et al., 2022) software suite.
3. An end-user study that employs these tools to collect data for an individualized characterization of eye gaze for control to provide further insights into the design of interfaces for systems that use eye gaze as input.
4. An evaluation and comparison of mental workload measures for suitability in monitoring workload while using an eye gaze interface.

In Section 2, we provide background on the related literature. In Section 3, we present the contributed virtual tasks designed for characterizing gaze movements during gaze-based control. In Section 4, we detail the data gathering pipeline, as well as the experimental setup and protocol. In Section 5, we present and discuss the results from the end-user study. In Section 6, we discuss the limitations of this work as well as avenues for future work, followed by conclusions in Section 7.

## 2. Background

In this section, we provide background on related literature on eye gaze for control, customization in interface design, and real-time measures for mental workload.

### 2.1. Eye gaze for control

Alternative control interfaces such as eye gaze trackers are gaining popularity with their increasing commercial availability and technological improvements. In addition to its use as a clinical control interface, eye gaze tracking has been increasingly adopted in the fields of human-computer interaction (HCI) and human-robot interaction (HRI) to evaluate the usability of interfaces (Cairns and Cox, 2008), as well as for control input in robot teleoperation and shared human-autonomy control contexts (Aronson and Admoni, 2020). Work in this latter area includes improving the interpretation of human control signals, as well as improving the control of assistive devices in conjunction with robot autonomy (Argall, 2018; Luo et al., 2021).

In the field of robotics, systems use eye gaze as an *explicit control input* – where voluntary eye movements are used to issue control commands and interact with the input space, usually a screen, to teleoperate robotic manipulators (Li et al., 2017), mobile robots (Carreto et al., 2018), and powered wheelchairs (Araujo et al., 2020). Eye gaze can also be used as an *implicit control input* to robotic systems, where user intention or goals are inferred from eye gaze data and used to provide control assistance (Stolzenwald and Mayol-Cuevas, 2019). Thus, eye gaze is a multi-interpretable interface, with both explicit and implicit ways to interpret the control input.

Using eye gaze for input presents challenges due to the dual role of vision in both perceiving the environment and issuing explicit control commands. This can lead to the “Midas touch” problem (Jacob, 1990); that is, the difficulty in differentiating whether a gaze movement is intended for interface operation (e.g., to control forward wheelchair movement) or simply for the purpose of perception (e.g., checking for cars before crossing a street). Proposed solutions include combining eye gaze with another input modality, which may not be viable for individuals with limited motor function, as well as implementing a dwell time requirement for control selection (Majaranta and Bulling, 2014). However, the latter approach may result in a perceived decrease in system responsiveness (Jacob, 1990). Hence, the improvement of paradigms using pure eye gaze input is of interest.

When used for powered wheelchair driving, many interfaces implement *zones* on the screen, where looking at a certain area of the screen activates a virtual button that issues a discrete control command for the wheelchair (ToltTechnologies, Lin et al., 2006). Like all discrete control interfaces, this presents challenges in controllability for the user, which can be further confounded by the “Midas touch” problem if simultaneously scanning the environment while issuing control commands results in unintended or jerky motion. In this work, we identify and analyze eye gaze characteristics during eye-based control with the aim of uncovering alternative ways of using the gaze signal, such as for simultaneous explicit and implicit control, as well as continuous control.

### 2.2. Measures for mental workload

To encourage long-term adoption, it is important for interactions with an eye gaze control interface to not be overly mentally taxing. Physiological (objective) measures, performance-based assessments, and self-reported (subjective) measures of MWL are commonly employed in the literature (Wilson, 2002).

Other common objective metrics focus on extracting physiological states through the use of psychophysiological measures to understand and quantify human emotional responses. In the field of robotics, psychophysiological measures index human emotional responses (Rani et al., 2002; Bethel et al., 2007; Tamantini et al., 2023a,b) and can be used to modulate human-robot interaction paradigms, for example during robot-aided rehabilitation (Tamantini et al., 2023a). The most common measurements include Electrodermal Activity (EDA), skin conductance or Galvanic skin response, heart rate (HR), respiration (RR intervals), EEG, and skin temperature (Bethel et al., 2007; Novak et al., 2010; Tamantini et al., 2023a,b).

#### 2.2.1. Heart rate and heart rate variability metrics

Both heart rate (HR) and heart rate variability (HRV) (Charles and Nixon, 2018) are well-established metrics to estimate MWL (Mulder and Mulder, 1981; Delliaux et al., 2019). HR is a physiological measure that reflects the rhythm of the heart’s contractions and the rate at which blood is pumped through the body. Different factors such as emotional state (e.g., mental workload) can significantly influence HR.

HRV measures the variation in the time interval between heartbeats over time. The heartbeats are known as inter-beat intervals (IBIs) or RR intervals – the time between successive R-peaks in an electrocardiogram. This measurement is used to draw inferences on the Autonomic Nervous System (ANS) linked to stress and MWL – the “flight or fight” response mode – as it can affect the force and contraction rate of the heart (Sztajzel, 2004; Ariansyah et al., 2018; Massaro and Pecchia, 2019).

Higher HR and lower HRV are linked with increased cognitive load and mental effort (Mulder and Mulder, 1981; Hjortskov et al., 2004; De Rivecourt et al., 2008; Delliaux et al., 2019) and index task difficulty (Gergelyfi et al., 2015). Metrics for HRV include time-domain, frequency-domain, and non-linear measures. Time-domain measures are classified according to the time duration of the measurement: (1) Ultra-short-term (UST, <5 min), (2) short-term (~5 min), and (3) 24-hr term (Shaffer and Ginsberg, 2017). The most common time-domain metric to measure HRV is the Root Mean Squared of Successive Differences (RMSSD) (Shaffer et al., 2014; Shaffer and Ginsberg, 2017).

#### 2.2.2. Gaze-based metrics

Numerous metrics computed from eye gaze measurements, such as gaze movement metrics and pupil-based metrics, have been proposed and shown to be effective at measuring MWL. In particular, pupillary response is shown to reflect mental activity, as well as a correlation between mental activity and task difficulty (Hess and Polt, 1964), wherein a larger pupil size reflects a higher MWL (Beatty, 1982). Changes in the duration of fixations and saccadic amplitudes from their respective means suggest focal processing (higher MWL than baseline) or ambient processing (lower MWL than baseline) (Krejtz et al., 2016). The Index of Pupillary Activity (IPA) (Duchowski et al., 2018) uses the frequency of pupil diameter oscillation as a metric for cognitive load. There has been extensive work in the use of gaze-based measures in monitoring the MWL of users in a human-machine interaction context – operating wearable assistive technologies (Marchand et al., 2021), driving (Marquart et al., 2015), and physical human-robot interaction (cobot operation) (Upasani et al., 2023). However, there has been limited research conducted in the realm of concurrently extracting MWL data from the gaze signal while also eliciting a control signal.

#### 2.2.3 NASA Task Load Index

Compared to the objective HRV measurements, the NASA Task Load Index (TLX) survey (Hart, 2006) is a subjective but well-established method to assess workload. The survey is designed to rate performance across six different categories and from these determine an overall workload rating: (1) Mental Demand, (2) Physical Demand, (3) Temporal Demand, (4) Performance, (5) Effort, and (6) Frustration.

### 2.3. The importance of customization

Input signals measured by interfaces are subject to interpretation, and discrepancies between a user’s intended control commands and those received by the machine can exist. User-specific calibration (software or physical) can help to address these discrepancies. Variability in physiology, behavior, and skill over time may result in a need for regular recalibration (Gillham et al., 2017).

In the domain of eye gaze control, the accuracy and usability of the system are highly dependent on good calibration of the system to the user (Jacob, 1990). However, the calibration of gaze-tracking systems often is described as tedious and difficult (Pfeuffer et al., 2013). In this work, we look at eye gaze characteristics on an individual basis in order to better inform alternative avenues for customization in eye-based control systems, in addition to standard calibration procedures.

## 3. Eye gaze characterization

Previous work that evaluates eye-gaze-based input systems for assistive devices has been application-oriented, with evaluations based on success in completing tasks *performed by the assistive device* (such as an application-specific GUI or powered wheelchair) which is controlled via eye gaze (Araujo et al., 2020; Sibert and Jacob, 2000). In this work, we are interested in *characterizing* eye-based control input in order to better inform the design of eye-gaze user interfaces for controlling assistive devices. For this reason, we want to collect general eye movement data that covers the spectrum of possibilities in using the eyes for input, rather than specific to a particular application (such as controlling a particular device or using a particular user interface). To do this, we present a *suite of application-independent tasks* intended to characterize eye gaze by isolating the features of interest in the eye gaze signal.

**Figure 1.**
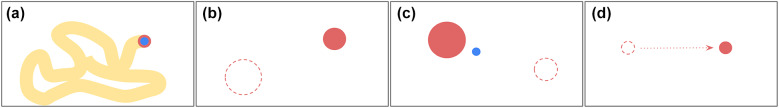
The screen-based tasks implemented in Unity2D. (a) The Painting Task. Participants control a blue cursor with the 5-min countdown indicated as a radially filling red outline. (b) The Focus Task without visual feedback on gaze position. Targets fade away as participants fixate on the target continuously for 2 s, and the next target appears. (c) The Focus Task with visual feedback on gaze position. As in (b), but now with a blue dot representing the gaze position, as measured by the eye gaze tracking system, for visual feedback. (d) The Tracking Task. Participants track moving targets on the screen. For panels (b–d), red-filled circles represent the current target. Dashed circles and arrows are provided for illustration purposes only, and represent the prior target (circles) and movement of targets (arrow).

Each gaze characterization task is designed to collect gaze data for specific types of gaze movement patterns and metrics during eye-based control. Gaze patterns and metrics of interest include saccades, fixations, and smooth pursuits, as these types of eye movement events are commonly used in gaze-based control interfaces (Majaranta and Bulling, 2014; Duchowski, 2017), and also expected to occur in the domain that we are interested in – assistive device control – as it involves observation of a dynamic environment (Sibert and Jacob, 2000). The eye gaze characterization tasks are screen-based tasks implemented in the Unity^®^ Engine (Unity Editor Version 2021.3.10f1) (Unity Technologies, 2021). An illustration of each of the screen-based tasks described is shown in Figure 1, with video included in the Supplementary Material, and is described as follows.

- ***Painting Task***
*Task description*: Eye gaze position controls a virtual paintbrush. Prior to the start of the task, the participant is instructed to fixate on the virtual paintbrush, initialized at the center of the screen. They are then asked to ‘paint’ the entire screen with their eyes within 5 min.

*Features of interest*: Distribution of eye gaze, distribution of fixations, and areas of visual neglect.

- ***Focus Task***
*Task description*: The participant views and fixates on 60 randomly appearing circular targets of varying sizes on the screen and is asked to maintain their gaze for 2 s. If 2 s of continuous fixation are not maintained by the end of 10 s, the target times out.

*Features of interest*: Saccades, fixations, and dwell time.

*Visual feedback*: Feedback on how much time remains of the 2 fixation requirement, via variations in opacity. A target becomes more transparent the longer the participant dwells on it, up to 2 s when the target fades completely and the next target appears. Two variants of the task exist: (1) The participant is not given any visual feedback of where their measured gaze is. (2) The participant is provided with this feedback, in the form of a moving dot on the screen.

- ***Tracking Task***
*Task description*: Twenty-four moving targets (circles) appear on the screen one after another. The participant is tasked with following the targets with their gaze. Targets move at a speed of approximately 15 degrees of visual angle per second.

*Features of interest*: Smooth pursuit.

*Visual feedback*: Feedback on the proximity of the participant’s measured gaze to the moving target, via a variation in the opacity of the target (more opacity indicating being closer to the target).

## 4. Methodology

This section outlines the study protocol and materials used in the study – including experimental setup and procedure, details regarding recruited participants, and the methods used for data analysis of workload using eye gaze and heart rate.

### 4.1. Participants

A total of 11 participants were recruited: 1 individual with ALS (62 yo, female) and 10 individuals with spinal cord injury (SCI) (41.7 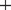 11.3 yo, (9 male and 1 female)). The SCI participants had varying injury levels (C5 to T12) and types (complete and incomplete). Specific details for each participant are provided in Table 1. All participants were screened for the ability to use an eye gaze tracker to interact with the screen via a simple test described in the experiment procedure. All participants gave their informed, signed consent to participate in the experiment, which was approved by Northwestern University’s Institutional Review Board (STU00217297).

**Table 1.** Details on recruited participants

Participant ID	Diagnosis	SCI classification	SCI level of injury
S01	ALS	–	–
S02	SCI	Unknown	T4
S03	SCI	Complete	T10
S04	SCI	Incomplete	C6–C7
S05	SCI	Complete	C5
S06	SCI	Complete	T4
S07	SCI	Complete	C5
S08	SCI	Incomplete	T12
S09	SCI	Complete	T12
S10	SCI	Complete	T-unknown
S11	SCI	Complete	T11–T12

### 4.2. Hardware and materials

**Figure 2.**
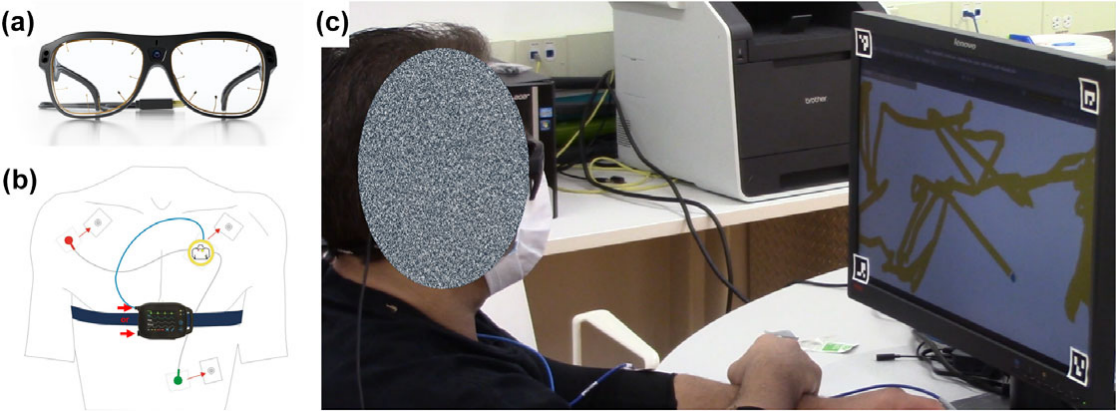
(a) Tobii Pro Glasses 3 (TPG3) head-mounted eye tracker (Tobii, 2021). (b) The 1-channel-ECG lead setup with the SOMNOtouch RESP cardiorespiratory screener (Polygraph) ECG Sensor (Somnomedics, 2018). (c) A participant performing the Painting Task. ArUco markers on the corners of the screen are used for transforming the gaze position in the scene to the gaze position on the screen.

The eye gaze interface consists of a head-mounted eye gaze tracker from Tobii (Danderyd Municipality, Sweden): Tobii Pro Glasses 3 (TPG3, 100 Hz gaze data rate, 1920p by 1080p @ 25 fps scene camera, Figure 2a). The TPG3 has been shown to yield more accurate eye-tracking results than the earlier (Tobii Pro Glasses 2) model used extensively in previous eye-tracking research (Onkhar et al., 2023).

The TPG3 uses corneal reflection dark pupil eye tracking, and provides scene camera video, gaze position in the scene camera frame, as well as 3D gaze position. Using the scene camera video, gaze position is transformed in real-time to position on the screen and the transformed signal is used as control input for study tasks. In addition to gaze position, the tracker also measures pupil diameter, which can be used as an additional source of implicit input to the control system. While not used in real-time for this study, pupil diameter data is analyzed post-hoc as an analog to measure cognitive load during the tasks.

To measure heart rate, the SOMNOtouch™ RESP Electrocardiogram (ECG) sensor from SOMNOmedic AG (Randersacker, Germany) is employed. The configuration includes the SOMNOtouch™ RESP, body strap, and the Docking Station. The DOMINOlight software is used for initialization, data transfer, and storage.

### 4.3. Eye gaze data collection pipeline

**Figure 3.**
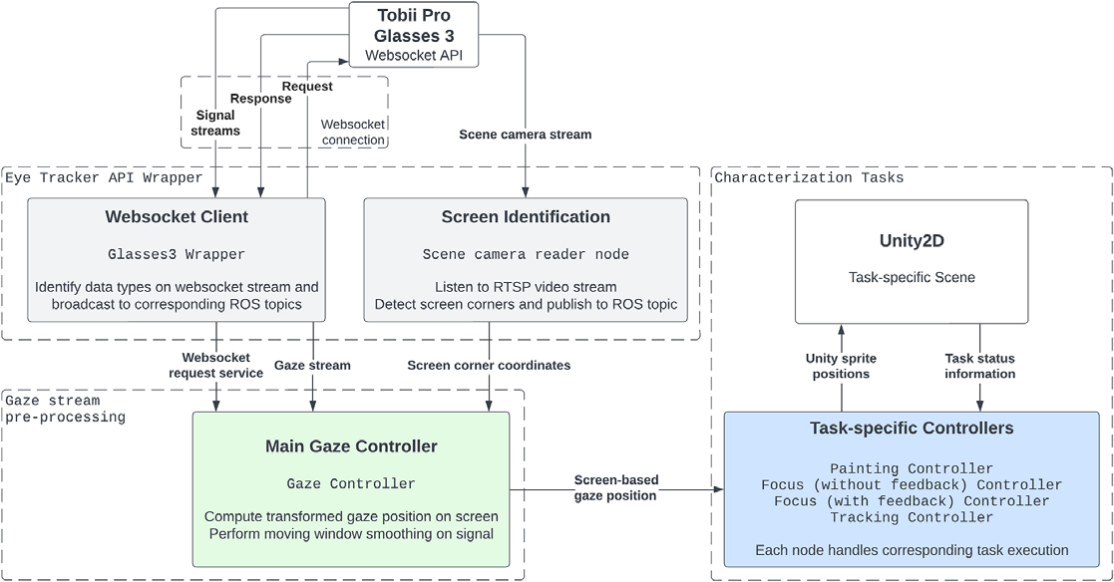
Schematic of our eye-gaze characterization system, implemented in ROS2. A gaze stream receiver and pre-processing node (green) receives input from eye tracker streamer nodes (gray) that are specific to a given eye tracking device and API. The preprocessing node publishes the processed **screen-based gaze position** 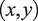 to task nodes (blue), which interact with Unity and handle task progress. All data is logged in ROS bagfiles for post-hoc analysis. Communication with Unity2D takes place over their provided ROS TCP Endpoint.

We integrated the application-independent eye gaze characterization tasks from Section 3 into a data collection pipeline outlined in Figure 3, and our code is publicly available on github (Loke, 2023). The provided code works out-of-the-box with the TPG3 hardware and API.

To facilitate portability, the data collection pipeline is implemented within the Robot Operating System (ROS) (Macenski et al., 2022), an open-source software suite used by millions across the world. Our contributed gaze characterization tasks will work with any eye tracker that streams the 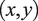 coordinates of gaze position on a 2D screen. To use the eye gaze characterization tasks in a data collection pipeline, the following software nodes are needed:

- An eye tracker-specific *streamer node(s)* that publishes data from the eye gaze tracker to ROS2. In Figure 3, the streaming nodes comprise the gray boxes that wrap the eye tracker API.
- A *receiver node* that operates on the eye tracker’s gaze data streams to handle real-time gaze stream *preprocessing* (such as moving-window average smoothing of the signal) and publishes the screen-based gaze position. In Figure 3, the receiving and preprocessing are handled by the Main Gaze Controller (green). In our implementation, the receiver node additionally handles the conversion from head-mounted scene camera gaze coordinates to screen-based gaze coordinates, which is necessary because the TPG3 tracks gaze in 3D (versus tracking on a screen).
- *Task controller nodes* (blue) that handle calculations relating to task progress and broadcast relevant data streams for task control to Unity®.

Communication between Unity2D and ROS2 takes place over the ROS TCP Endpoint provided by Unity (Unity Technologies, 2022).

### 4.4. Experiment procedure

#### 4.4.1. Setup

A participant first is fitted with the SOMNOtouch™ RESP Electrocardiogram (ECG) sensor. ECG electrodes are connected to an ECG sensor and placed at three locations on the upper body: he left and right sides of the torso below the collarbone and above the fifth intercostal space of the left side of the body (Figure 2b). A fingertip pulse oximeter is placed on the participant’s left index finger. The participant’s blood pressure readings are recorded using the Welch Allyn Home Blood Pressure Monitor with SureBP Technology (Ontario, Canada) for the purposes of calibrating the ECG sensor through the SOMNOtouch data recording and processing software.

Following this, the participant is seated at a 60 cm viewing distance from a 55.9 cm (22 in.) 1600p by 900p screen and fitted with the head-mounted TPG3 eye gaze tracker (Figure 2c). For those who wear glasses, corrective lenses matching their glasses power are added to the TPG3. The eye gaze tracker then is calibrated using the TPG3’s standard built-in calibration procedure available through the WebSocket API. To calibrate, the participant is asked to fixate on two concentric black circles on a white background, without moving their head. After calibration, a simple sanity check test is conducted to verify that the participant is able to use the eye gaze tracker to issue the required control inputs: they are asked to look at the four corners of the screen and the recorded signal is verified to match expected values. After this, the participant proceeds with the study tasks.

#### 4.4.2. Tasks

Each task consists of a training phase and a testing phase. In the training phase, the participant is introduced to and familiarized with the task via a shortened version of the full-length task. The testing phase then consists of executing the given task for the prescribed duration or number of repetitions (as described in Section 3). The presentation of tasks is fixed across participants: (1) Painting Task, (2) Focus Task *without* gaze position feedback, (3) Focus Task *with* gaze position feedback, and (4) Tracking Task. After each task, participants are asked to fill out a NASA Task Load Index (TLX) (Hart, 2006) survey. After completing all 4 tasks, the participant’s blood pressure is measured again.

### 4.5. Analysis methods

We analyze gaze position data to identify eye movement events during task execution for eye gaze characterization, as well as pupil diameter and heart rate data to compute metrics of mental workload.

#### 4.5.1. Gaze position data

Eye movement event detection is an established field of research with various algorithmic approaches proposed in the literature (Startsev and Zemblys, 2022). The process of eye movement event detection often involves a preprocessing step, that denoises the gaze signal by way of filtering, followed by the classification of gaze signal segments as different types of eye movements such as *saccades*, *fixations*, and *(smooth) pursuits.* This classification, also known as event detection, can be done by parametric or non-parametric methods, wherein parametric methods make use of velocity, acceleration, and temporal thresholds to identify types of eye movements, and non-parametric methods often make use of machine learning techniques to extract inherent patterns in the gaze signal.

Salvucci and Goldberg (2000) propose a taxonomy for describing and comparing event detection algorithms, wherein algorithms can be differentiated by their spatial (velocity-based, dispersion-based, or area-based) and temporal (duration sensitivity and local adaptivity) characteristics. In addition, they identify five representative algorithms in eye movement event detection and present a qualitative comparison of their characteristics, concluding that *velocity-thresholding-based* algorithms have the greatest potential for real-time use (fastest) while yielding comparable accuracy to other methods with minimal parameter tuning required.

For this work, we therefore use the open-source REMoDNaV eye movement classifier implemented in Python (Dar et al., 2021) to do offline (post-hoc) classification of gaze movements. REMoDNaV implements an improved version of the velocity-based algorithm proposed by Nyström and Holmqvist (2010) and is capable of classifying fixations, saccades, post-saccadic oscillations, and smooth pursuit events. REMoDNaV was chosen for the following reasons: (1) It is designed to work with both static and dynamic stimuli, both of which are present within our gaze characterization tasks. (2) It is shown to perform reasonably well on lower-quality eye movement data, which we might expect to encounter in assistive device control applications in the wild. (3) It is a velocity-based (rule-based) algorithm and hence can be used out of the box without ground-truth labels or further model training, unlike for example deep-learning-based methods (such as Startsev et al. (2018), Zemblys et al. (2018)).

**Statistical analysis.** Reported statistical significance between groups is determined by a non-parametric independent samples t-test (Mann–Whitney U-test). Where not reported, differences in groups are not statistically significant. Where specified, eye movement distance is reported in degrees of visual angle (DVA), computed using screen size, viewing distance, and screen resolution. (In our experimental setup, 1 pixel 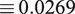 DVA.)

#### 4.5.2. Pupil diameter data

Changes in pupil diameter are shown to be an indicator of changing MWL. Pupil diameter data measured by an eye tracker in millimeters (mm) needs to be appropriately preprocessed to remove invalid pupil diameter samples. To do so, we follow the guidelines outlined by Kret and Sjak-Shie (2019). The steps taken in pre-processing are:

1. Remove *size outliers*: Samples outside the feasible range of 1.5–9 mm.
2. Remove *dilation speed outliers*: Samples with disproportionately large absolute pupil diameter change relative to adjacent samples.
3. Remove *time series gaps*: Samples within 50 ms of gaps in the pupil diameter time series that are larger than 75 ms.

The primary metric computed from pupil diameter data in this work is the *Change in Pupil Diameter (CPD)* (Krejtz et al., 2018). A given participant’s CPD is computed relative to their baseline pupil diameter. The baseline pupil diameter measurement is taken from the 2 s prior to starting the first task, as participants are assumed to be at neutral MWL during that time. CPD is then computed over a single task, as well as over 10 s rolling (5 s overlapping) windows, relative to the baseline pupil diameter.

#### 4.5.3. Heart rate data

Given the time duration of our tasks (<5 min), we compute and report the UST RMSSD HRV metric in this study. Measurements for HR are extracted from the recorded RR intervals using the known hyperbolic relationship: HR x RR Interval = 60000 (Goldberger et al., 2014). RR Interval – also referred to as the Inter-Beat Interval (IBI) – is the peak-to-peak measurement of time (in milliseconds) between each heartbeat, and is calculated on the DOMINOlight software following the transfer of data.

Both HR and HRV are inspected for artifacts (missing or spurious beats) that can significantly distort time-domain measurements (Peltola, 2012; Shaffer and Ginsberg, 2017). Affected RR intervals are manually edited (Shaffer and Combatalade, 2013) via removal of ectopic beats and arrhythmia detected by the SOMNOtouch RESP sensor. Participants’ RR intervals are filtered for any arrhythmic beats.

**Statistical analysis.** We compute both offline metrics and online time series measures for MWL. Offline MWL metrics are computed on the entire duration of each task. We assess reliability and agreement between the three MWL metrics (CPD, HR, RMSSD) using the Intraclass Correlation Coefficient (ICC, single-rater two-way fixed effects model) (Koo and Li, 2016), a common measure of interrater reliability. Before calculating the ICC, MWL metrics are normalized by their minimum and maximum values for each participant in order to compare the relative trends within each metric. Additionally, negative RMSSD is used as it is negatively correlated with MWL.

Online MWL measures are computed for each task, in 10-second rolling windows (5-s overlap) of the raw data time series where each measure is computed over each window. We use Bland–Altman analysis (Martin Bland and Altman, 1986) to assess the agreement between measures, and Dynamic Time Warping (DTW, Giorgino, 2009) to assess the similarity between their time series. A normalized similarity measure is computed from the cumulative distance between the two time series being compared after aligning them via DTW.

## 5. Results and discussion

This section presents the results and insights from end-user interaction with our gaze characterization tasks and pipeline. We discuss results relating to eye movement characteristics and metrics during each of the tasks and present a comparison of MWL metrics to assess the potential utility of eye-tracked measures as indicators of MWL.

### 5.1. Eye gaze characterization

Here we present the eye movement characteristics and metrics gathered while completing our eye-gaze characterization tasks. We direct our discussion to the context of possible use cases for powered wheelchair driving control. A pain point in current eye-based input methods for driving powered wheelchairs is the lack of commercial availability of continuous control (Araujo et al., 2020), and so our later analyses focus in particular on continuous inputs.

#### 5.1.1. Spatial layout of a user interface

During the Painting Task, participants are encouraged to explore the space of the screen as much as possible. To ensure users are able to issue intended commands, the user interface should be designed in a way that allows users to operate in the area of the screen that they are most comfortable and skilled in. We are thus interested in how the spatial distributions indicate regions of positive or negative preference for eye gaze signal provision. To quantify this, we propose an index of *displacement from the uniform coverage* – a user’s baseline coverage of a region of the screen. We define a unit of uniform coverage 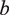 as the total number of samples divided by the number of grid cells (the uniform coverage distribution). With this index, zero displacement maps to uniform coverage, while positive and negative displacement from zero displacement scale proportionally, from 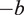 displacement (no gaze data in that grid cell) and saturating at 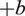 displacement.

**Figure 4.**
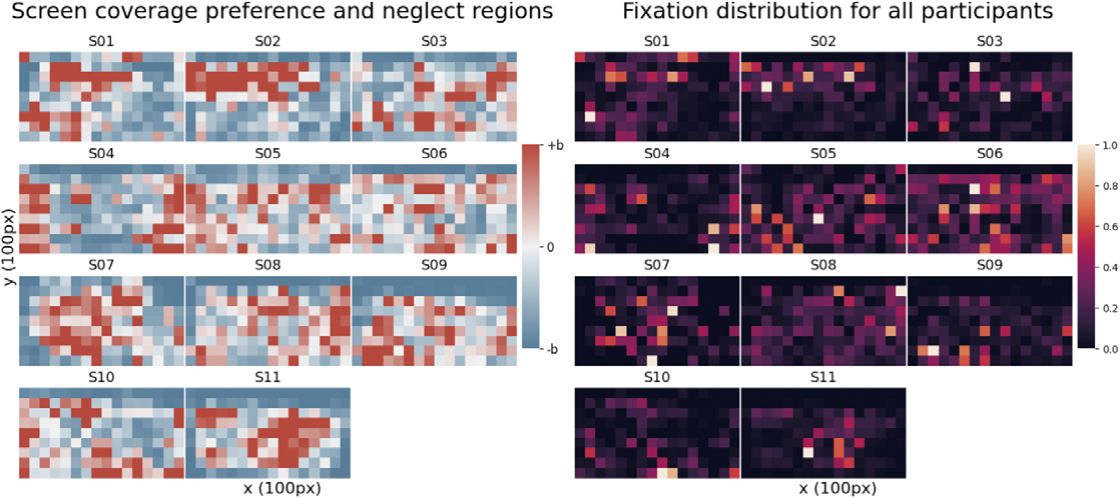
Painting Task, Left: Index of displacement from uniform screen coverage. Each grid represents a 100px by 100px area. The intensities of the heatmap range from 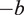 displacement to 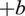 displacement from uniform coverage. Zero displacement is a user’s baseline screen coverage. Right: Distribution of **fixations** across the screen. Each grid represents a 100px by 100px area. The intensities of the heatmap are normalized on a scale from 0 to 1 for each participant, and normalization is done relative to the counts in the most frequently occurring 100px by 100px area in the heatmap. Higher intensities indicate regions of greater demonstrated ability to dwell.

Figure 4 (left) presents a visual representation of the screen coverage displacement index, where ‘neutral’ (white) areas correspond to zero displacement, ‘hot’ (red) areas correspond to positive displacement, and ‘cool’ (blue) areas correspond to negative displacement. Figure 4 (right) presents the spatial distributions of fixations. We observe a large inter-participant variability for both the index of displacement from uniform coverage, as well as the spatial distribution of fixations. From the presented regions of preference and neglect, a user such as S02, for instance, would likely benefit from a user interface that expects eye gaze input in the upper half region of the screen, while S04 would likely benefit from an interface that does not expect eye gaze input in the center of the screen.

#### 5.1.2. Virtual button sizes

**Figure 5.**
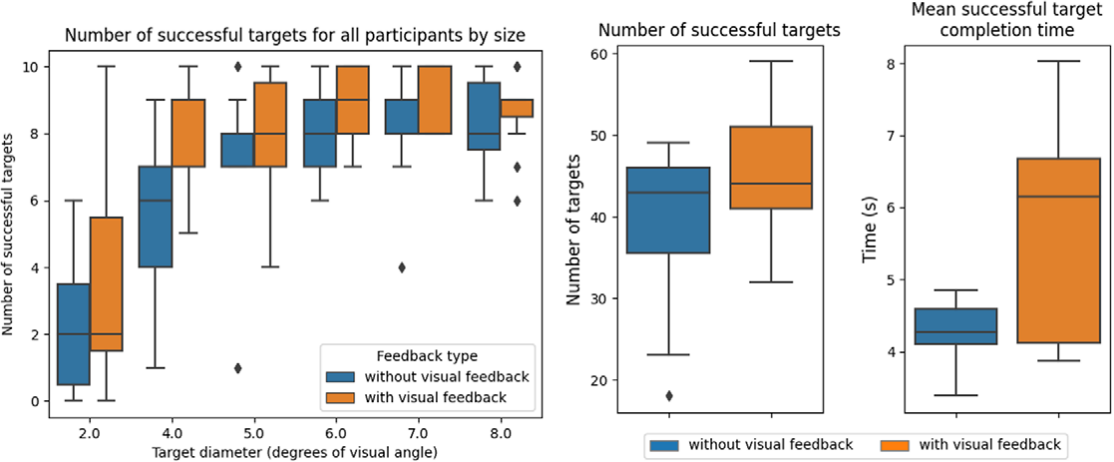
Target success for the *Focus Task.* Left: Number of successful targets, grouped by target size, for all participants and under both feedback conditions. We observe an approximate minimum button size for which most participants are able to successfully fixate (of 4.0 (5.0) DVA with (without) visual feedback). Right: Number of successful targets (left) and mean time taken to complete successful targets (right) for all participants, comparing without and with visual feedback for measured gaze position. We observe a trade-off between success and completion time when visual feedback is present.

In Figure 5 (left), the number of successful targets (out of 10 targets total) is shown for the Focus Task, grouped by target size and both *without* visual feedback and *with* visual feedback. In Table 2, we present the first and second derivatives of the curve fit (asymptotic exponential) to the median number of successful targets at each size.

**Table 2.** The first and second derivatives of the curve fit the median number of successful targets shown in Figure 5 (left)

Target diameter	Without feedback		With feedback	
(DVA)	1st derivative	2nd derivative	1st derivative	2nd derivative
2	3.29	−1.66	27.40	−113.78
4	1.20	−0.61	0.01	−0.03
5	0.73	−0.37	0.00	0.00
6	0.44	−0.22	0.00	0.00
7	0.27	−0.13	0.00	0.00
8	0.16	−0.08	0.00	0.00

From the results, we see that most participants are able to successfully fixate for 2 s consecutively on targets that have a diameter larger than 5 degrees of visual angle (DVA) and that the slope at 5.0 DVA drops below 1.0, accompanied by a significantly reduced magnitude of the second derivative between 4.0 to 5.0 DVA, and 5.0 to 6.0 DVA, indicating plateau onset.

These results suggest that, in general, virtual on-screen icons that require dwell to activate should not be smaller than 5.0 DVA in size. Customization of icon size can also be done according to users’ success at such a task. When visual feedback is provided, participants are able to successfully achieve targets with an even smaller diameter (4.0 DVA), suggesting that on-screen icons may be even smaller if users have visual feedback.

#### 5.1.3. Visual feedback for gaze location

Figure 5 (right) shows the overall target completion success rate and mean target completion time for the Focus Task, under both visual feedback conditions. In terms of task success, we see that participants perform better overall with visual feedback than without visual feedback. However, participants tend to take longer to successfully complete targets with visual feedback. Due to the limited sample size, statistical significance is not observed in these results; however, their general trend suggests the potential presence of a difference.

Anecdotal vocalizations from participants while completing this task suggest a split in preference and performance between the use cases of with and without visual feedback. Participants who prefer having gaze position feedback cite that they are better able to understand the system, and thus adapt their eye movements to successfully complete the task; these participants generally perform better *with* feedback. On the other hand, participants who perform better *without* gaze position feedback state that the visual feedback is distracting and results in ‘cursor chasing’ due to inconsistencies between the perceived gaze location and the displayed measured gaze location (Vickers et al., 2008). This suggests the need to (1) better design the gaze location feedback modality, and (2) allow users to toggle on/off the gaze cursor, which is already an option in many gaze-based control systems (Microsoft, 2024).

#### 5.1.4. Continuous pursuit motion

**Figure 6.**
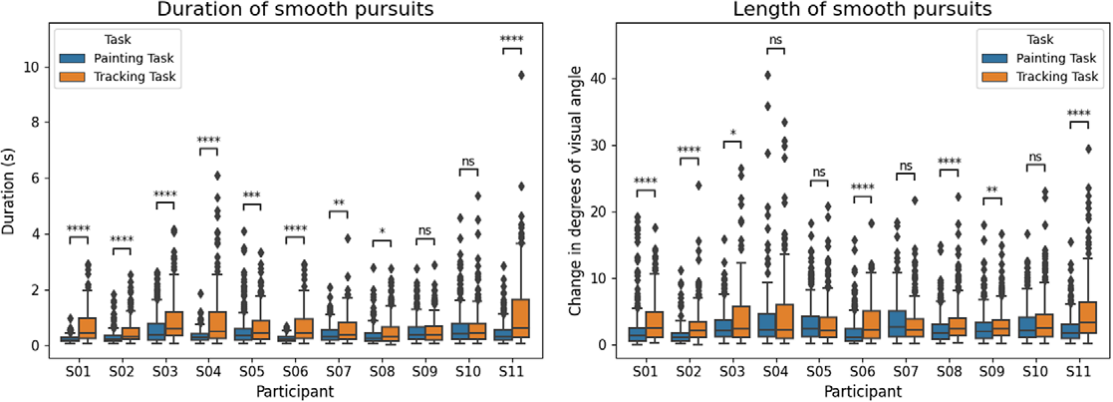
Comparison of smooth pursuits between the *Painting Task* and *Tracking Task.* Left: Duration of smooth pursuit segments, for all participants. Right: Length (magnitude) of smooth pursuit segments, in degrees of visual angle, for all participants. Significant differences between the two tasks are seen for most participants, suggesting the possibility of the length and duration of smooth pursuits to differentiate between using the eyes for observation versus control.

Continuous inputs can likely be achieved by pursuit motions, which are slower and more controlled than saccadic motions. Figure 6 shows the distributions of the time duration and magnitude (in degrees of visual angle) of smooth pursuits. In general, smooth pursuit segments during the Tracking Task tend to be longer in mean duration and of a greater magnitude than those during the Painting Task. This difference is statistically significant ( 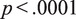 ) for both duration and magnitude over the whole population, and also statistically significant in 10 of 11 participants for duration and/or magnitude.

The significant difference in smooth pursuit duration and magnitude is a preliminary result that points to the possibility of extracting continuous control signals from eye movements in a safe and intelligent manner. The differences in smooth pursuit metrics likely are attributable to the Tracking Task providing a moving target for the eyes to focus on, while the Painting Task requires intentional motion of the eye to move the cursor on the screen.

Thus, for some individuals, it may be possible to differentiate whether the eyes are being used for control input versus being used to scan the environment, based on the duration of smooth pursuits over a short time history. This may be used to more intelligently overcome the ‘Midas touch’ problem.

Another interpretation of this result for interface design could be to use the median of an individual’s length of smooth pursuit segments during the Painting Task to inform the control resolution of a virtual joystick on a screen – individuals able to more frequently provide larger distances of smooth pursuit when controlling a cursor with their eyes might be able to control a virtual joystick over a larger visual area, offering higher control resolution.

### 5.2. Mental workload during task completion

We investigate both offline (post-hoc) and online (real-time) workload measurements. Offline measures of workload allow us to identify the types of eye motions which may be more taxing for individuals, to inform interface design. Online measures open up the possibility of using real-time, on-the-fly estimates of MWL to inform the customization of control, such as the modulation of control assistance in a shared human-autonomy control paradigm (Luo et al., 2021).

HR, RMSSD, and CPD are examined both as a single value computed over the entire duration of the task (offline), and as time series data (online). Only 9 out of the 11 participants had valid ECG data and hence only results from these 9 participants are included in the discussion involving physiological measures.

#### 5.2.1. Task-level workload assessment

**Figure 7.**
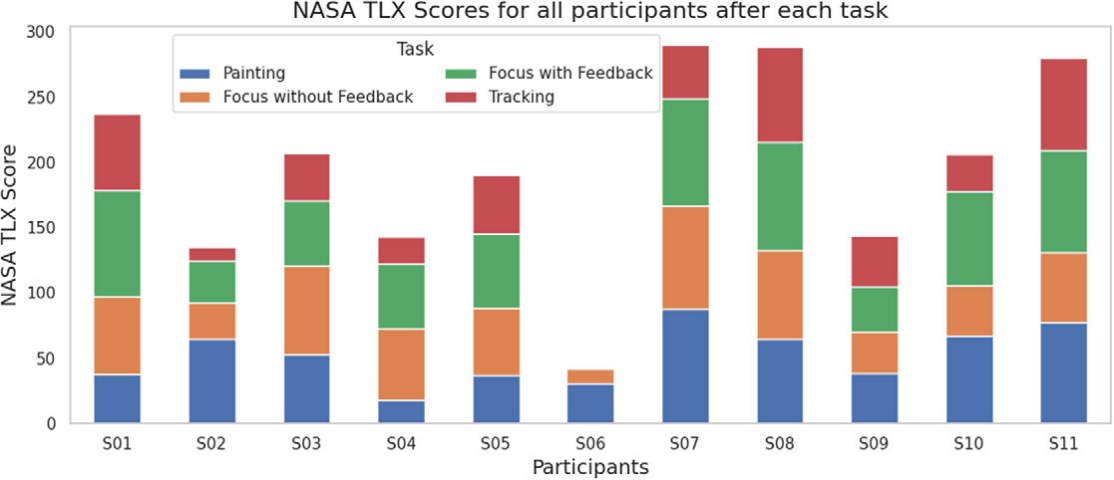
NASA TLX Scores for all participants (S01–S11), stacked by task. We observe large inter-participant variability in perceived overall MWL, as well as in the relative MWL required of each task.

Figure 7 shows the NASA TLX scores (out of 100 for each task) for all participants after completing each of the tasks. A high TLX score correlates with more cognitive workload. From the results, there is a lot of variability in the perceived MWL across participants, with scores ranging from zero workload to near maximal. For a given participant, we further observe instances of consistency across tasks (e.g., consistently high cognitive load for all tasks) as well as marked variability (e.g., some tasks rated low and others high).

Most participants report lower TLX scores for the Tracking Task (4th task). Since the Tracking Task requires participants to follow a moving target (reactionary) as opposed to controlling a cursor (as in the Painting Task), it makes sense that participants would find it less mentally taxing.

Anecdotally, participants reported that one of the largest difficulties they faced with using the eye gaze tracker was a perceived steep learning curve. The TLX scores across the tasks may have captured learning effects in some participants. In particular, S02 and S07 generally report decreased levels of workload from the first to the last task, while S06 self-reported zero workload for the 3rd and 4th tasks, citing that they had now learned and understood the system.

#### 5.2.2. Comparison of offline metrics

While subjective metrics are important to learn about a person’s perceived mental workload towards a task, combining them with objective metrics is often best practice (Hoffman and Zhao, 2020). Our results contribute to the ongoing discussion regarding the reliability and validity of subjective versus objective metrics in assessing MWL. In addition to the TLX score, we also compute each participant’s HR, RMSSD, and CPD over the entire duration of each task. The computed Intraclass Correlation Coefficient (ICC) of selected metrics for the population of 9 participants is presented in Table 3, with results for each participant presented in the Appendix, Table A1.

**Table 3.** Intraclass Correlation Coefficient (ICC) to estimate interrater consistency of the MWL metrics, over all participants

Metric combinations		ICC
All		0.310
Physiological only		0.452
CPD	HR	0.669
TLX	CPD	0.423
CPD	RMSSD	0.356
HR	RMSSD	0.338
TLX	HR	0.117
TLX	RMSSD	−0.017

**Figure 8.**
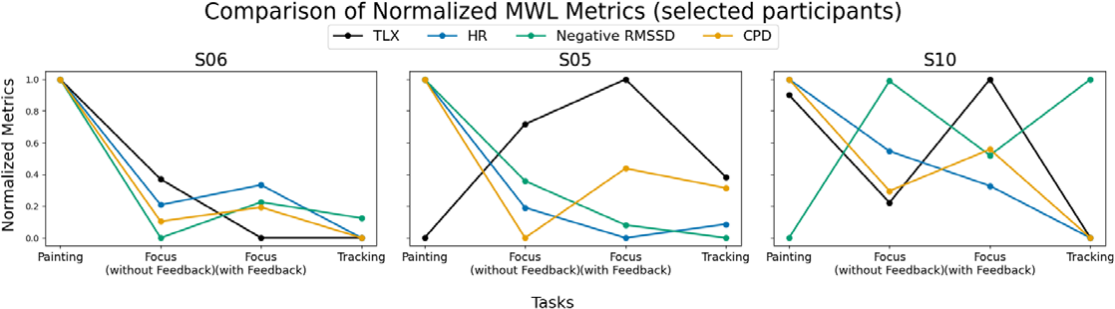
Illustrative comparison of NASA TLX, HR, RMSSD (negative), and CPD over tasks for three participants. We observe instances of agreement across all measures (left), varying agreement/disagreement between physiological and subjective measures (middle), and no real trend between the measures (right). For the purposes of visualization and ICC calculation, all four metrics are normalized by their respective minimum and maximum values for each participant. The participants shown are representative of the variability in agreement between measures. Data from other participants is omitted to keep the presentation clear and concise.

In general, we see a range of reliability and agreement between the combinations of the four metrics for different participants, as illustrated in Figure 8. Averaged over all participants, we observe a general trend of agreement (albeit poor, ICC < 0.5) between all measures of MWL, with however stronger agreement between the physiological measures. Most (7 of 9) individual participants also follow this general trend (Appendix). When comparing the physiological and subjective measures, we observe the most agreement between TLX and CPD (in the average, and 7 of 9 participants), and weak disagreement between TLX and RMSSD (in the average, and 6 of 9 participants). CPD also is strongly correlated with HR (on average, and 8 of 9 participants).

These findings suggest that it is feasible to use CPD to assess participants’ perceived cognitive load during task execution, and hence gaze interface use. In contrast, the suitability of heart rate (HR) to assess perceived workload appears to vary across participants but appears to be superior to that of heart rate variability (RMSSD).

Our findings support related work that demonstrates agreement between subjective (NASA TLX) and objective (task completion time and eye gaze activity) measurements of MWL during eye gaze tasks (Chen et al., 2011). This related work also reports that subjective metrics are the most accurate method and the most often used for the evaluation of task difficulty design. While our findings suggest a more complicated relationship between the two types of measurements, we do also see a general agreement between them.

#### 5.2.3. Comparison of online measures

**Figure 9.**
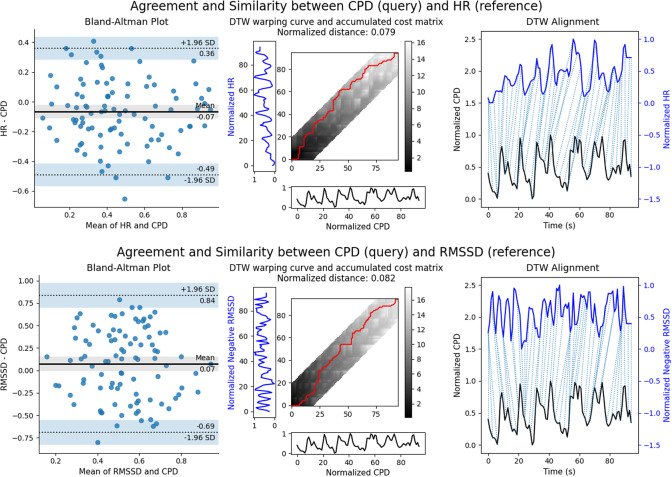
Visual comparison of agreement and similarity between CPD and HR (top), and CPD and RMSSD (bottom) for S11 during the Focus with Feedback Task. Left: Bland–Altman plot of agreement with limits of agreement (dashed lines) and mean bias (solid, black line). Middle: DTW optimal warp path in red, with 20% Sakoe-Chiba window constraint shown in the grey band. Right: DTW alignment showing matched points in the time series. We observe that a large majority of points lie within the LoA of the Bland–Altman plot when comparing CPD to both HR and RMSSD, as well as alignment in peaks of the CPD, HR, and RMSSD time series.

Time series of HR, RMSSD, and CPD are computed post-hoc for each task. In order to compare these measures quantitatively, each measure is normalized based on the minimum and maximum values in each time series. An example visualization of the results of Bland–Altman analysis and DTW comparing CPD (known as the query) against well-established metrics HR and RMSSD (known as references) is presented in Figure 9, for a single participant (S11) and task (Focus with Feedback). The same analysis was done for all 9 participants and all tasks with complete MWL metric data, and the results are summarized in Table 4. Detailed results are provided in the Appendix, Tables A2 and A3.

**Table 4.** Summary of Bland–Altman analysis and DTW Similarity aggregated over all participants

Metric combinations	% of points within limits of agreement (LoA)	DTW similarity score
HR and CPD	94.7%	78.2%
RMSSD and CPD	95.4%	70.1%

We observe that a large majority of the points in the Bland–Altman analysis for CPD compared with both HR and RMSSD lie within the 95% Limits of Agreement (LoA). The LoA indicates the range within which 95% of the differences between the query and reference metrics are expected to fall, demonstrating a 95% confidence that these metrics agree. DTW similarity scores are also above 70%, with CPD exhibiting higher similarity with HR than RMSSD. Hence, on a time series scale, CPD appears to offer information similar to that of HR and RMSSD.

This suggests that, in addition to offline determinations of workload, it might also be feasible to use CPD for online on-the-fly computations of workload. The potential utility of on-the-fly estimates of workload is broad, ranging from feedback for caregivers to input that modulates assistive device control. The caveat, however, is that CPD computation requires performing regular baseline measurements to ensure the computed measure accurately reflects the current state of the user.

## 6. Limitations and future work

The study presented in this work is subject to several limitations. A small sample size of participants makes it challenging to establish statistical significance, and the fixed task order for all participants may have influenced the results through learning or fatigue effects, which can bias TLX scores. Additionally, the gaze characterization tasks developed for this study lack validation in accurately measuring the intended gaze characteristics, and the study was conducted under specific experimental conditions and may not be representative of eye tracking in real-world applications. Future work could address these limitations by modifying the study protocol to randomize task order, expanding the participant pool, conducting validation studies to demonstrate the reliability and accuracy of these tasks, and varying the study environment to better mimic real-world scenarios.

Although we offer some conclusions regarding the provision of continuous inputs to gaze-based interfaces by leveraging individuals’ smooth pursuit characteristics, our proposed tasks do not fully characterize the difference between intentional control input and environment scanning or observation. Future work could include a user-controlled version of the Tracking Task, where users control a gaze cursor to take the same path as in the Tracking Task, in order to collect data that directly characterizes the difference in smooth pursuit metrics under intentional control input versus environment observation.

Despite the manual removal of artifacts from ECG signals to preserve the integrity of HRV metrics, there is scope for further work to automate this process – thereby decreasing potential human error associated with manual inspection. Additionally, incorporating baselines, calculated from HR calibration data recorded for each participant at the beginning of a session, could enhance the analysis by tailoring it to the unique physiological characteristics of each participant.

This study focused on the analysis of MWL; however, additional research could include an analysis of stress and MWL through the use of different subjective measures such as the Short Stress State Questionnaire, which measures task-induced stress (Helton and Näswall, 2015). A comparison of different subjective measures could inform better practices for implementing tasks and determining whether a user is under cognitive workload.

## 7. Conclusion

In this article, we have presented an open-source data collection pipeline to characterize eye gaze for device control. This pipeline consists of a suite of screen-based assessment and data-gathering tasks to characterize eye gaze movements during eye-based control and a system to use an eye gaze tracker for real-time control input with ROS. The aim of this system is to probe an individual’s ability to interact with a screen using eye gaze and a variety of eye movements – namely, saccadic motions, fixations, and smooth pursuits. Probing these characteristics is the first step towards allowing us to identify parameters for designing a customizable and adaptive control interface design.

Towards this end, we conducted a study in which candidate end-users of an eye-gaze interface operated our eye-gaze characterization system while collecting heart rate data for analyzing mental workload during each task. We have presented insights from the data with regards to the design of an explicit continuous eye gaze tracking user interface – in particular, that the spatial layout of interfaces as well as button sizes should be customized to each user’s gaze characteristics, and that users’ smooth pursuit characteristics may be used to differentiate controlled gaze input from environment scanning. We also analyzed both offline and online metrics for measuring mental workload when providing eye-based control inputs. Results examining the agreement and reliability between different measurements support the feasibility of using the gaze signal to simultaneously extract control signals and workload metrics. Our future work will present users with different eye input user interface designs based on these insights and assess usability.

## Supporting information

Loke et al. supplementary material Loke et al. supplementary material

## Data Availability

The data that support the findings of this study are available from the corresponding author, L.L., upon reasonable request. ROS Galactic (Ubuntu 20.04) code and Unity^®^ Engine (Unity Editor Version 2021.3.10f1) package are available at: https://github.com/argallab/eyegaze_characterization_tasks.

## Footnotes

1
https://github.com/argallab/eyegaze_characterization_tasks

## Supplementary material

The supplementary material for this article can be found at http://doi.org/10.1017/wtc.2024.27.

## Appendix: Additional data from the comparison of MWL measures

In Table A1, we present the results of the ICC computation between the measures for each individual participant. This demonstrates the range of reliability and agreement between the combinations of the four metrics across different participants.

**Table A1.** Intraclass Correlation Coefficient (ICC) to estimate interrater consistency of the MWL metrics aggregated for each participant

Participant	All	Physiological	TLX	TLX	TLX	CPD	CPD	HR
			HR	RMSSD	CPD	HR	RMSSD	RMSSD
All	0.310	0.452	0.117	−0.017	0.423	0.669	0.356	0.338
S01	0.098	0.352	−0.117	−0.280	−0.180	0.945	0.183	−0.133
S02	0.398	0.313	0.848	−0.299	0.908	0.798	0.017	0.110
S03	0.686	0.914	0.317	−0.711	0.318	0.964	0.876	0.904
S05	0.058	0.834	−0.839	0.691	−0.669	0.807	0.710	0.972
S06	0.927	0.971	0.894	0.841	0.918	0.987	0.979	0.948
S08	0.263	0.602	−0.279	−0.524	0.610	0.529	0.285	0.962
S09	−0.079	−0.308	−0.567	0.613	0.397	−0.526	0.668	−0.983
S10	−0.071	−0.326	0.563	−0.840	0.849	0.891	−0.955	−0.773
S11	0.505	0.842	0.411	−0.040	0.563	0.835	0.731	0.965

In Tables A2 and A3, we present the results of the Bland–Altman and DTW analysis for each participant for a single task: Focus with Feedback. We report the following:

- *Bias*: The mean of the differences between the query and reference metrics.
- *95% Limits of agreement (LoA)*: The interval within which 95% of differences between measurements are expected to fall. If the differences between the metrics consistently fall within these limits, it suggests good agreement between the two metrics, with 95% confidence. It also shows the range in error of query metric (CPD) compared to reference metric (RMSSD or HR).
- *Percentage of points within LoA*: The average percentage of acceptable points within the LoA of all tasks for each participant.
- *DTW Similarity Score*: After the optimal mapping is found, the remaining cumulative distance between the two-time series can be obtained and used to compute a normalized similarity metric between the two-time series. Since the maximum value of the distance between the two-time series is 1 at each point after normalization, the length of the time series can be used as the normalizer.

**Table A2.** HR and CPD: Systematic Bias, 95% LoA, and percentage of points within the LoA for each participant for the Focus with Feedback Task

Participant	Bias	95% LoA	% of points within	DTW similarity score
			Limits of agreement (LoA)	
S01	−0.16	−0.70 to 0.38	96.1%	84.2%
S02	−0.07	−0.49 to 0.36	93.7%	84.2%
S03	0.31	−0.25 to 0.86	94.1%	79.2%
S05	0.16	−0.51 to 0.83	97.9%	79.3%
S06	0.14	−0.32 to 0.61	93.9%	82.9%
S08	0.32	−0.07 to 0.71	93.7%	72.9%
S09	−0.22	−0.71 to 0.26	94.8%	76.9%
S10	−0.31	−0.60 to −0.03	94.1%	72.0%
S11	0.02	−0.60 to 0.64	96.0%	85.8%

**Table A3.** RMSSD and CPD: Systematic Bias, 95% LoA, percentage of points within the LoA, and DTW Similarity Score for each participant for the Focus with Feedback Task

Participant	Bias	95% LoA	% of points within	DTW similarity score
			Limits of agreement (LoA)	
S01	0.24	−0.52 to 0.99	96.1%	74.4%
S02	0.07	−0.69 to 0.84	98.9%	83.5%
S03	0.35	−0.30 to 1.00	97.0%	72.0%
S05	0.05	−0.53 to 0.63	91.7%	83.3%
S06	0.18	−0.31 to 0.68	95.9%	82.0%
S08	0.22	−0.26 to 0.69	92.8%	82.5%
S09	0.37	−0.22 to 0.95	93.1%	63.8%
S10	0.45	0.04 to 0.87	92.6%	48.3%
S11	0.23	−0.39 to 0.85	95.2%	78.3%
